# Cochlear Mechanics Are Preserved After Inner Ear Delivery of Gold Nanoparticles

**DOI:** 10.3390/ijms26010126

**Published:** 2024-12-26

**Authors:** Dorothy W. Pan, Jinkyung Kim, Patricia M. Quiñones, Anthony J. Ricci, Brian E. Applegate, John S. Oghalai

**Affiliations:** 1 Caruso Department of Otolaryngology-Head and Neck Surgery, University of Southern California, Los Angeles, CA 90033, USA; 2 Department of Otolaryngology, Stanford University School of Medicine, Stanford, CA 94304, USA; 3 Department of Otolaryngology, Washington University School of Medicine, St. Louis, MO 63110, USA; 4 Alfred Mann Department of Biomedical Engineering, University of Southern California, Los Angeles, CA 90089, USA

**Keywords:** inner ear drug delivery, nanoparticles, optical coherence tomography, cochlear mechanics

## Abstract

Novel therapeutic delivery systems and delivery methods to the inner ear are necessary to treat hearing loss and inner ear disorders. However, numerous barriers exist to therapeutic delivery into the bone-encased and immune-privileged environment of the inner ear and cochlea, which makes treating inner ear disorders challenging. Nanoparticles (NPs) are a type of therapeutic delivery system that can be engineered for multiple purposes, and posterior semicircular canal (PSCC) infusion is a method to directly deposit them into the cochlea. We sought to assess PSCC infusion of gold NPs into the cochlea, including the NPs’ distribution and effect on cochlear mechanics. We performed optical coherence tomography (OCT) imaging to monitor PSCC infusion of gold NPs into the cochlear chambers. OCT imaging demonstrated that the infusion specifically targeted the perilymphatic spaces within the cochlea. We assessed cochlear mechanics by using OCT vibrometry to measure sound-evoked movements of the basilar membrane. We found no changes in cochlear mechanics between measurements at baseline, after the PSCC canalostomy, immediately after the infusion, and 1 h after the infusion of gold NPs (*p* > 0.05, paired *t*-test). These findings validate the PSCC infusion approach for perfusing the cochlear perilymphatic space with a nanoparticle delivery system. Thus, PSCC infusion of nanoparticles is a feasible therapeutic delivery technique for treating inner ear disorders while preserving residual cochlear function.

## 1. Introduction

Inner ear disorders resulting in hearing loss are a problem worldwide, and the prevalence is increasing as the population ages [1,2,3]. Sensorineural hearing loss is a debilitating condition that results in social isolation, cognitive decline, and even increased mortality [4,5,6,7,8,9]. Treatment of sensorineural hearing loss currently remains limited to auditory prosthetic devices such as hearing aids and cochlear implants.

Developing therapies to treat hearing loss remains difficult because many barriers to therapeutic delivery into the inner ear exist [10,11,12,13,14]. Systemic delivery is limited by the blood labyrinth barrier, akin to the blood brain barrier, which is permeable only to select molecules due to tight junctions between endothelial cells and pericytes and perivascular macrophages surrounding the basement membrane of the endothelial cells [15,16,17,18,19]. The cochlea is encased in dense otic capsule bone, in a location deep within the temporal bone, which also complicates local therapeutic delivery. Intratympanic injection can be done in clinic by an otolaryngologist, but this has limited penetration into the cochlea due to the presence of the round window membrane, which causes a 1000-fold decrease in drug concentration between the middle ear and inner ear, as well as a gradient in drug concentration from the base decreasing to the apex [20].

Other local drug delivery methods to the inner ear include infusion directly through the round window membrane or into the lateral or posterior semicircular canal. These both require an outpatient surgical procedure but are much more efficient at delivering therapeutics into the inner ear as they directly access the inner ear fluid chambers. Round window infusion is accessible with a surgical approach through the ear canal. The infusion initially enters the scala tympani and carries a risk of sensorineural hearing loss due to direct infusion into the cochlea [21]. This technique has shown efficacy with partial restoration of hearing in one genetic form of human hearing loss [22,23]. Infusion into the lateral or posterior semicircular canal is another technique used to deliver therapeutics directly into the inner ear space. Lateral semicircular canal infusion has been proposed for use in early clinical trials. Posterior semicircular canal (PSCC) infusion has mainly been used in rodent models such as mice [24,25,26,27]. Either lateral or posterior semicircular canal infusion is easily translatable to human patients as it requires a simple mastoidectomy, which is a routine outpatient surgery done for chronic ear infections or cochlear implantation. Though PSCC infusion of therapeutics has been performed in animal models and shown gene transduction in the inner ear, the immediate distribution of injected material within the cochlea after PSCC infusion has not been well studied. Recent studies prove that PSCC infusion initially distributes throughout the cochlear perilymphatic space. Thus, the basolateral surfaces of hair cells and supporting cells within the organ of Corti would be exposed to the perfusate [28]. As the semicircular canal infusion technique does not directly access the cochlea, an advantage of this technique is a decreased risk of sensorineural hearing loss, and work done on mouse models show minimal impact on vestibular function [21,28,29].

The delivery systems, or delivery vehicles, under investigation for inner ear drug and gene therapy are vast and varied [30,31]. Viral vectors are typically used to deliver genetic material in animal models, most commonly adeno-associated virus [32,33]. They are minimally pathogenic and efficient at transduction in the inner ear; however, they have limited capacity for genetic material, 4.7 kb for a single AAV vector, and are limited in the capacity to deliver therapeutics other than in the form of nucleic acids [34]. One alternative to viral vectors are nanoparticles. Nanoparticles are not pathogenic and can be made with a wide variety of materials. They are versatile in their ability to deliver small-molecule therapeutics to macromolecular therapeutics, including nucleic acids and proteins [14,31,35,36]. They can be targeted to specific cell types and be delivered intracellularly via receptor-mediated endocytosis [37,38,39]. For example, using ex vivo inner ear rodent animal models, gold nanoparticles have been shown to be targeted to hair cells and deliver Myosin XVa plasmid constructs intracellularly so that the protein can be expressed at the appropriate location, the tips of hair cell stereocilia [40,41]. However, one potential downside to using nanoparticles in the cochlea is their mass. When they settle, there may be adverse effects on the vibratory properties of the organ of Corti.

To address this point, we characterized the distribution of nanoparticles after PSCC infusion as well as their effect on cochlear mechanics. We chose to use gold nanoparticles rather than other types of nanoparticles because they have a high refractive index and can be detected using optical coherence tomography imaging [42]. Furthermore, they have intrinsic two photon fluorescence properties [43,44,45,46,47,48] and can be readily functionalized with fluorophores or conjugated with targeting agents or therapeutics including peptides, proteins, or nucleic acids [39,49,50]. Finally, they are among the heaviest of nanoparticles and thus provide a rigorous test for mass loading of the organ of Corti.

## 2. Results

### 2.1. Gold Nanoparticles Synthesis and Characterization

**Figure 1:**
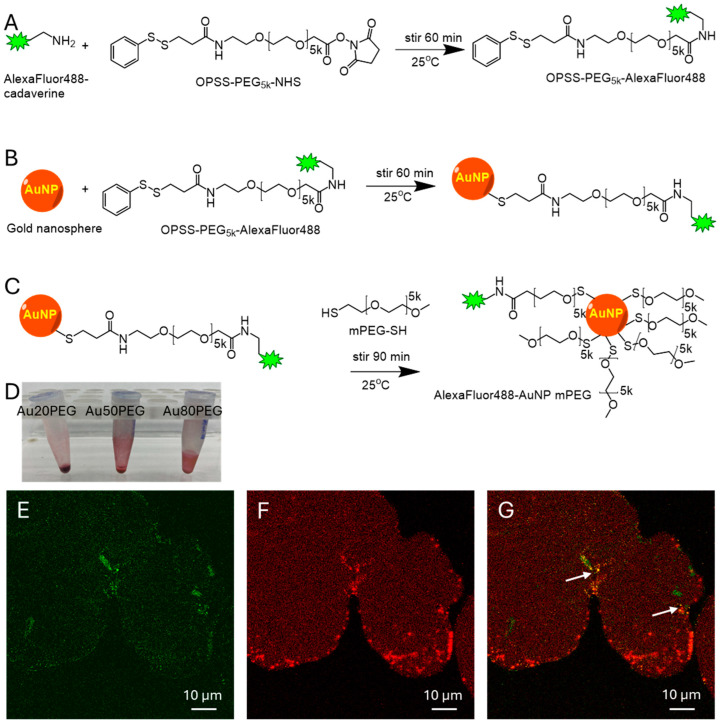
Synthesis of functionalized and stabilized gold nanoparticles lead to characteristic color and visibility on two-photon imaging. (**A**) Synthetic scheme for OPSS-PEG5k-AlexaFluor488, a step to link the AlexaFluor488 fluorophore to the gold nanoparticle core. (**B**) Synthetic scheme showing the attachment of AlexaFluor488 fluorophore to the gold nanoparticle core via a polyethylene glycol 5 kDa linker. (**C**) Saturating the remaining binding sites on the gold nanoparticle core with polyethylene glycol to completely coat the gold nanoparticle to make a formulation that is stable in physiological salt solutions and does not aggregate. (**D**) Photo showing the dark pink to brown color of the concentrated Au20PEG, Au50PEG, and Au80PEG nanoparticles in artificial perilymph. Confocal microscopy of a drop of Au50PEG on a glass slide showing the (**E**) AlexaFluor488 fluorescence in the green channel, (**F**) gold two-photon fluorescence in the red channel, and (**G**) merged signal of the green and red channels with arrows pointing to clusters of nanoparticles.

Spherical gold nanoparticle cores aggregate in physiological salt solutions, as previously demonstrated in the literature [51]. Therefore, stabilized gold nanoparticles functionalized with AlexaFluor 488 fluorophore (Figure 1A) and a polyethylene glycol 5 kDa coating (Figure 1B,C) were synthesized using 20 nm, 50 nm, and 80 nm spherical gold nanoparticle cores to result in Au20PEG, Au50PEG, and Au80PEG nanoparticles, which are stable without aggregation in physiologic salt solutions such as artificial perilymph. The final hydrodynamic diameter, polydispersity index (variation in nanoparticle size), and zeta potential (surface charge of the nanoparticle) were measured using dynamic light scattering and shown in Table 1. The hydrodynamic diameter of Au20PEG was 52.3 ± 0.7 nm (mean ± standard error), Au50PEG was 83.1 ± 2.0 nm, and Au80PEG was 101.8 ± 1.0 nm, respectively. The polydispersity index was narrow, at 0.268 for Au20PEG, 0.164 for Au50PEG, and 0.158 for Au80PEG. The zeta potential was slightly negative at −10.53 ± 2.06 mV (mean ± standard error) for Au20PEG, −12.46 ± 1.27 mV for Au50PEG, and −1.71 ± 1.40 mV for Au80PEG. These values are comparable to similar gold nanoparticles synthesized previously [39,49]. The nanoparticles were exchanged into artificial perilymph solution and concentrated to a calculated concentration of 7.8 × 10^13^ particles/mL for Au20PEG, 3.7 × 10^12^ particles/mL for Au50PEG, and 1.1 × 10^12^ particles/mL for Au80PEG, with a gold mass concentration of 5.3 mg/mL. These gold nanoparticles have a dark pink to brown hue at these high concentrations (Figure 1D). Two-photon microscopy of a drop of nanoparticles on a slide at 20× magnification with excitation wavelength 930 nm showed fluorescence in the green channel (emission 494–529 nm) from the AlexaFluor488 functionalization (Figure 1E), as well as in the red channel (Emission 588–643 nm) (Figure 1F) for gold nanoparticles’ intrinsic 2-photon fluorescent properties [43,44,45,46,47,48], therefore having signal in both the green and red channels shown by the arrows (Figure 1G).

### 2.2. OCT of the Cochlea During Gold Nanoparticle Infusion via the Posterior Semicircular Canal Demonstrates Perilymphatic Compartment Filling and Perfusion of the Entire Cochlear Duct

**Figure 2:**
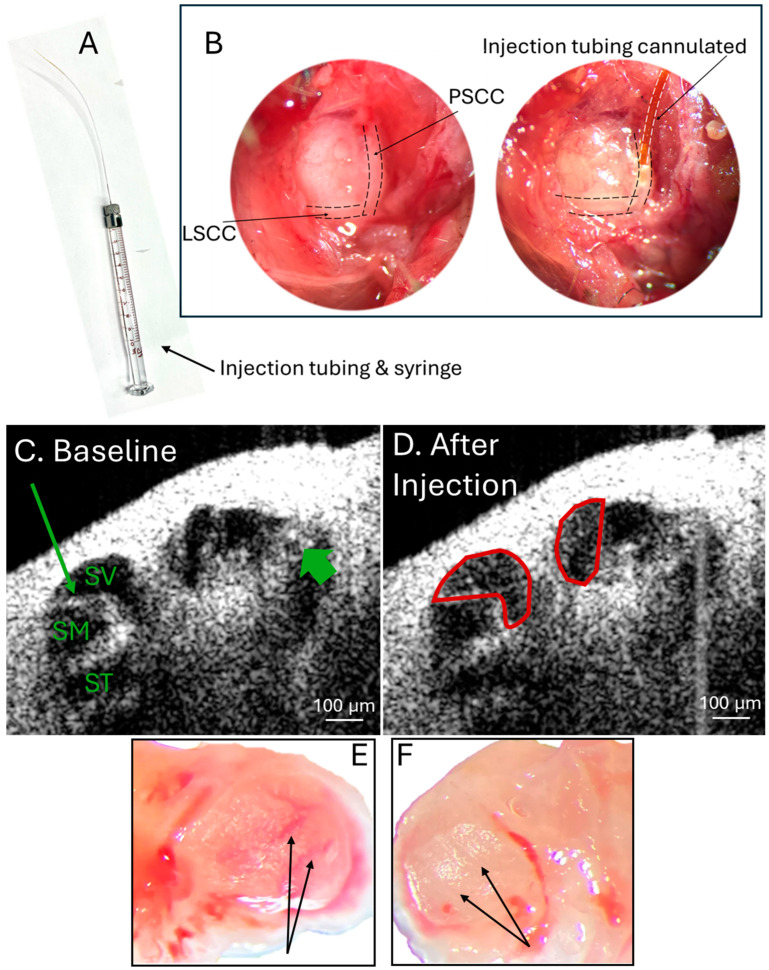
PSCC infusion of gold nanoparticles can be visualized with OCT in vivo and in the cochlear duct of excised cochlea. (**A**) Infusion tubing on a 10 µL gastight Hamilton syringe for PSCC infusion. (**B**) Photo of the posterior and lateral semicircular canal exposed in a mouse under anesthesia (left), and with the infusion tubing cannulated into the posterior semicircular canal (right). (**C**) OCT image of the apical turn of the wild-type CBA/CaJ mouse cochlea showing the basilar membrane (thick arrow), Reissner’s membrane (thin long arrow), scala vestibuli (SV), scala media (SM), and scala tympani (ST) at baseline after opening the bulla to expose the cochlea. (**D**) OCT image of the same apical turn location of the cochlea after Au50PEG infusion via the PSCC showing increased signal in the perilymphatic spaces especially of the scala vestibuli (chamber outlined in red) from the gold nanoparticles. (**E**) Photo of an excised left cochlea that had been perfused with Au50NP via the PSCC showing a pink hue in the cochlear duct (arrows) from the nanoparticle color. (**F**) Photo of the contralateral excised right cochlea that had not undergone nanoparticle infusion, which does not demonstrate the pink hue in the cochlear duct (arrows).

Posterior semicircular canal infusion of 1 µL of the synthesized gold nanoparticles was performed in 7–10-week male and female wild-type and normal hearing CBA/CaJ mice. This was performed as has been described previously [26,28] with infusion tubing attached to a gastight 10 µL Hamilton syringe (Figure 2A) filled with gold nanoparticles in artificial perilymph solution cannulated into the PSCC (Figure 2B). Continuous OCT cross-sectional images of the cochlea were recorded during the 0.5 µL/min nanoparticle infusion to create a video showing nanoparticles entering and filling the entire perilymphatic compartment, including the scala tympani and scala vestibuli (Supplementary Video S1). This data is consistent with what we previously reported and the reported perilymph space of 0.6–1.7 µL in mice [28,29,52]. Still images were taken before (Figure 2C) and after (Figure 2D) nanoparticle infusion showing an increased density, which represents the gold nanoparticles present in the perilymphatic spaces, particularly in the scala vestibuli (Figure 2D). Furthermore, visualization of the otic capsule itself shows the pink hue of the gold nanoparticles along multiple cochlear turns in the cochlear duct of the left cochlea that was perfused (Figure 2E, arrows), which is absent from the non-perfused contralateral right cochlea (Figure 2F, arrows).

### 2.3. Cochlear Mechanics in Wild-Type CBA/CaJ Mice Are Preserved After PSCC Infusion of Gold NPs

Next, we assessed whether the vibratory properties of the organ of Corti were affected by the gold NPs. We measured vibratory responses from the basilar membrane of the cochlear apical turn. The characteristic frequency, or frequency of maximal vibration to a low stimulus intensity, at the locations we studied ranged between 7–10 kHz at baseline.

**Figure 3:**
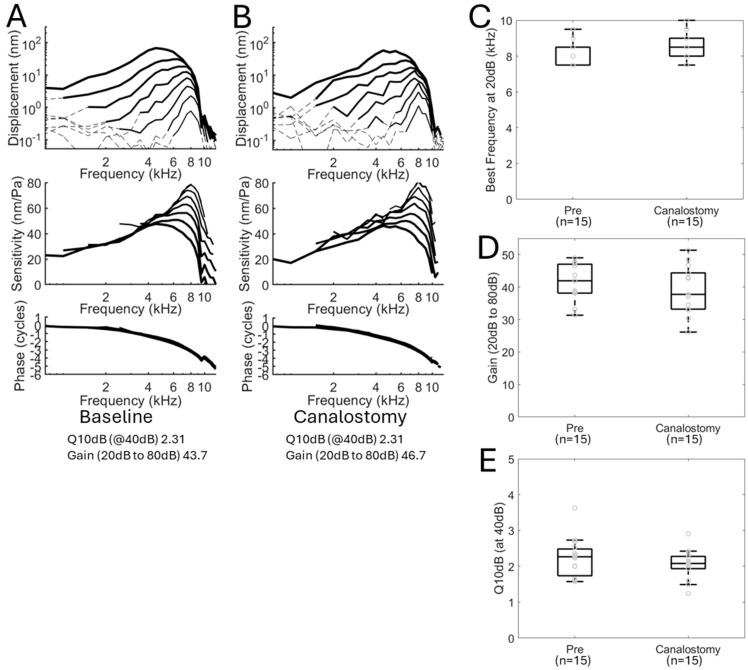
Cochlear mechanics are unchanged at baseline and after canalostomy with infusion tubing insertion into the PSCC. (**A**) Displacement of the basilar membrane, cochlear sensitivity, and phase from an apical turn location with characteristic frequency of 8 kHz in a wild-type CBA/CaJ mouse at baseline. (**B**) Displacement of the basilar membrane, cochlear sensitivity, and phase at the same cochlea location of the same mouse after performing a canalostomy and inserting the infusion tubing into the PSCC, but prior to infusion of nanoparticles. The tuning curves and phase plots show minimal changes to cochlear mechanics after canalostomy and tube insertion. (**C**) Best frequency of the basilar membrane motion, calculated at a 20 dB sound stimulus, from the apical cochlea location at baseline (Pre) and after canalostomy (Canalostomy) in *n =* 15 wild-type CBA/CaJ mice. (**D**) Cochlear gain between 20 dB and 80 dB sound stimulus showing no significant difference at baseline and after canalostomy in *n =* 15 wild-type CBA/CaJ mice. (**E**) Q10dB, calculated measurement of cochlear tuning sharpness at 40 dB sound stimulus, showing no significant difference at baseline and after canalostomy in *n =* 15 wild-type CBA/CaJ mice. Dotted lines in (**A**,**B**) indicate data below the noise floor. Circles in (**C**–**E**) indicate the values for each mouse.

First, we looked at whether differences existed between the baseline condition and after the canalostomy and sealing the infusion tubing in the canalostomy but prior to infusion of any material. Vibratory responses, including the displacement, the sensitivity (the displacement divided by the sound intensity), and the phase, are shown for one representative mouse (Figure 3A,B). There were no obvious differences in cochlear mechanics between the baseline and post-canalostomy conditions. We measured responses from 15 mice (seven females, eight males). To quantify differences between the baseline and post-canalostomy conditions, we then calculated the best frequency (the frequency of maximal vibration), the gain between 20 dB SPL and 80 dB SPL, and the sharpness of tuning using a stimulus intensity of 40 dB SPL (Q_10dB_, frequency of maximal vibration divided by the bandwidth 10 dB down from the peak) at baseline and after canalostomy for each mouse and found no differences. The best frequency at baseline was 8.3 ± 0.7 kHz (mean ± standard deviation) and after canalostomy was 8.5 ± 0.8 kHz, which was not significantly different (*p* = 0.3, paired *t*-test). Gain between 20 dB SPL and 80 dB SPL was 41.0 ± 6.0 at baseline and 39.1 ± 7.4 after canalostomy, which was also not significantly different (*p* = 0.4, paired *t*-test). Finally, the Q_10dB_ also did not change significantly between baseline 2.23 ± 0.54 and after canalostomy 2.05 ± 0.5 (*p* = 0.15, paired *t*-test) (Figure 3).

**Figure 4:**
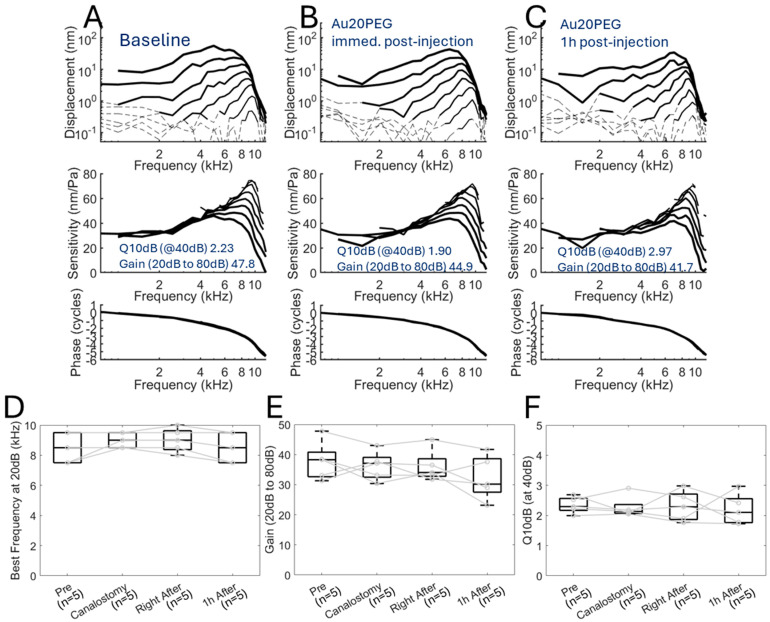
Cochlear mechanics are unchanged after Au20PG NP infusion. Displacement of the basilar membrane, cochlear sensitivity, and phase at the apical turn with characteristic frequency 9 kHz of a representative wild-type CBA/CaJ mouse at (**A**) baseline, (**B**) immediately after Au20PEG infusion, and (**C**) 1 h after Au20PEG infusion showing no significant change in cochlear mechanics after Au20PEG infusion. (**D**) Best frequency of the basilar membrane, (**E**) cochlear gain between 20 dB and 80dB sound stimulus, and (**F**) Q10 dB at 40 dB sound stimulus for a cohort of *n =* 5 mice showing no significant differences at baseline (Pre), after canalostomy (Canalostomy), right after, and 1 h after Au20PEG NP infusion. Dotted lines in (**A**–**C**) indicate data below the noise floor. Circles with connecting lines in (**D**–**F**) indicate the values for each mouse.

**Figure 5:**
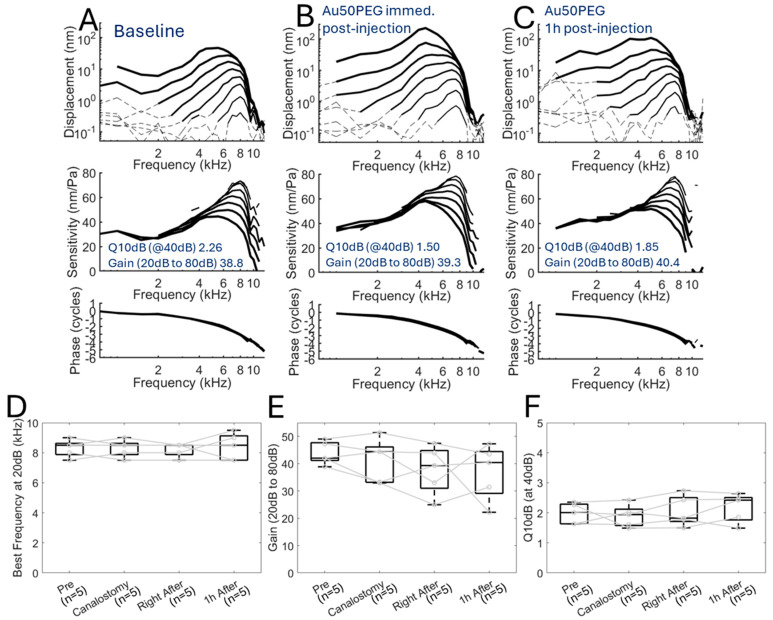
Cochlear mechanics are unchanged after Au50PEG NP infusion. Displacement of the basilar membrane, cochlear sensitivity, and phase at the apical turn with characteristic frequency 8 kHz of a representative wild-type CBA/CaJ mouse at (**A**) baseline, (**B**) immediately after Au50PEG infusion, and (**C**) 1 h after Au50PEG infusion showing no significant change in cochlear mechanics after Au50PEG infusion. (**D**) Best frequency of the basilar membrane, (**E**) cochlear gain between 20 dB and 80 dB sound stimulus, and (**F**) Q10 dB at 40 dB sound stimulus for a cohort of *n =* 5 mice showing no significant differences at baseline (Pre), after canalostomy (Canalostomy), right after, and 1 h after Au50PEG NP infusion. Dotted lines in (**A**–**C**) indicate data below the noise floor. Circles with connecting lines in (**D**–**F**) indicate the values for each mouse.

**Figure 6:**
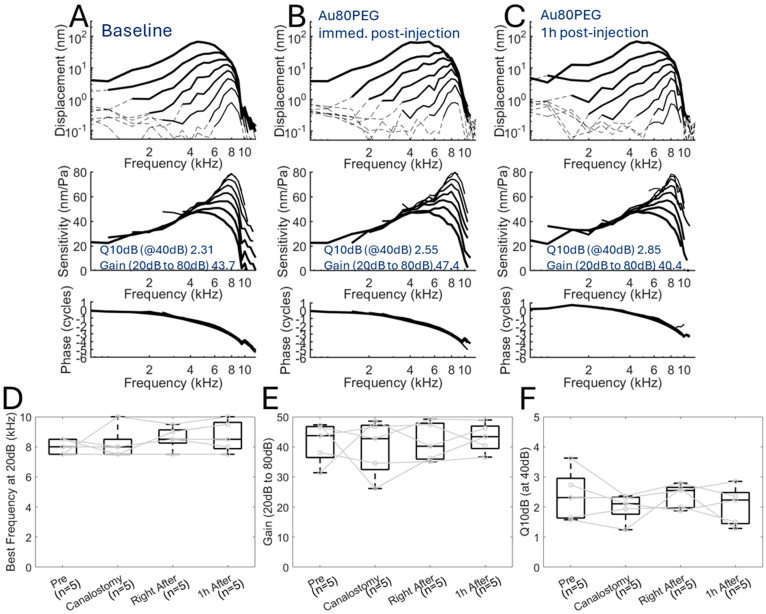
Cochlear mechanics are unchanged after Au80PEG NP infusion. Displacement of the basilar membrane, cochlear sensitivity, and phase at the apical turn with characteristic frequency 8 kHz of a representative wild-type CBA/CaJ mouse at (**A**) baseline, (**B**) immediately after Au80PEG infusion, and (**C**) 1 h after Au80PEG infusion showing no significant change in cochlear mechanics after Au80PEG infusion. (**D**) Best frequency of the basilar membrane, (**E**) cochlear gain between 20 dB and 80 dB sound stimulus, and (**F**) Q10 dB at 40 dB sound stimulus for a cohort of *n =* 5 mice showing no significant differences at baseline (Pre), after canalostomy (Canalostomy), right after, and 1 h after Au80PEG NP infusion. Dotted lines in (**A**–**C**) indicate data below the noise floor. Circles with connecting lines in (**D**–**F**) indicate the values for each mouse.

We then measured cochlear mechanics after infusion of one of the three different-sized nanoparticle formulations, Au20PEG, Au50PEG, and Au80PEG, to see whether infusing the nanoparticles would change cochlear mechanics and more specifically, whether the nanoparticle size causes any appreciable effect on cochlear mechanics. The vibratory responses in representative mice with infusion of either Au20PEG (Figure 4A–C), Au50PEG (Figure 5A–C), or Au80PEG (Figure 6A–C) are shown at baseline, immediately after nanoparticle infusion and 1 h after infusion. No obvious differences exist between these conditions for any size nanoparticle injected. Calculated measures of cochlear mechanics at baseline, after canalostomy, immediately after nanoparticle infusion, and 1 h after nanoparticle infusion included best frequency, gain between 20 dB SPL and 80 dB SPL, and Q_10dB_ at 40 dB SPL. The mean ± standard deviation for each of these calculated measures for a total of *n =* 5 mice per nanoparticle formulation are shown in Table 2. The calculated measures at each time point were compared to baseline and were not significantly different (paired *t*-test). Box plots of these results with points for each mouse in the group are shown for the Au20PEG (Figure 4D–F), Au50PEG (Figure 5D–F), and Au80PEG (Figure 6D–F). As no significant differences in any of these measures were seen between baseline, after canalostomy, immediately after nanoparticle infusion, or 1 h after nanoparticle infusion for a total of *n =* 5 mice per nanoparticle formulation, these data demonstrate that slow infusion of nanoparticles at 0.5 µL/min via the PSCC do not affect or change cochlear mechanics as the sharpness of tuning and gain did not change even immediately after PSCC infusion regardless of nanoparticle size.

It is interesting that the mass of the infused gold nanoparticles does not change cochlear mechanics. The infused gold mass in 1 µL for each nanoparticle formulation is 0.0053 mg. Based on morphological studies of the mouse cochlea, the basilar membrane has a length of approximately 6 mm, width of 139 µm, and thickness of 13 µm [53], or a volume of 9.98 nL [54]. An assumption that tissue density is approximately water density of 1 g/mL [55] estimates the basilar membrane mass to be 0.01 mg. As the gold nanoparticles are not visualized in the perilymphatic space by 1 h after infusion, they can be assumed to have settled. If you assume that the infused gold mass adheres to the basilar membrane, this would increase basilar membrane mass by 33%, a substantial change that would certainly alter cochlear tuning properties. Since this did not happen, it is possible that much of the gold mass adheres to non-vibratory structures such as the osseous spiral lamina or endosteum of the perilymphatic duct. Another factor that could explain the lack of change in cochlear mechanics after gold nanoparticle infusion is that although previous experimental results and theoretical models show that organ of Corti and basilar membrane mass does affect cochlear mechanics, mass changes are decreased towards the apex [56]. Furthermore, fluid density within the cochlea may play a larger role on cochlear mechanics than basilar membrane mass [57].

### 2.4. Optical Coherence Microscopy Combined with Two-Photon Imaging Shows Delivery of Gold Nanoparticles into the Cochlea Inner Hair Cell Region

**Figure 7:**
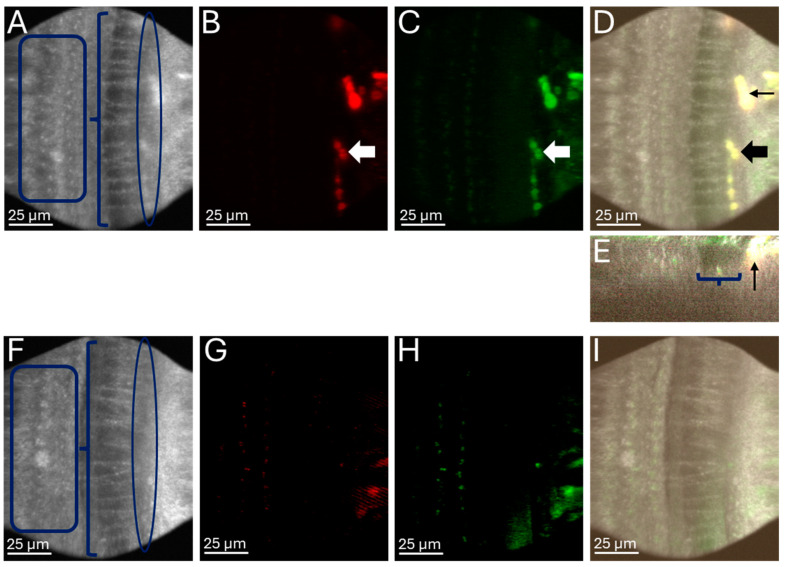
Optical coherence microscopy combined with two-photon fluorescence imaging shows Au50PEG nanoparticles reach the organ of Corti inner hair cell region. (**A**) Optical coherence microscopy image showing the outer hair cell region (rectangle), nerve fibers in the tunnel of Corti (bracket), and inner hair cell region (oval). (**B**) Two-photon fluorescence maximum Z projection from the red channel and (**C**) green channel, with (**D**) merged fluorescence signal overlaid on optical coherence microscopy image in a wild-type CBA/CaJ mouse 7 h after Au50PEG NP infusion via the PSCC showing fluorescent signal from the nanoparticles in the inner hair cell region (thick and thin arrows). (**E**) XZ view at the cross-section of the thin arrow in (**D**), which shows the area of the tunnel of Corti (bracket) and the fluorescence signal at the edge of the tunnel of Corti in the inner hair cell region (thin arrow). (**F**) Optical coherence microscopy image showing the outer hair cell region (rectangle), nerve fibers in the tunnel of Corti (bracket), and inner hair cell region (oval) with (**G**) two-photon overlay of the red channel and (**H**) green channel as well as the (**I**) merged two-photon fluorescence over the optical coherence microscopy image in a wild-type CBA/CaJ mouse control that had not had infusion of nanoparticles showing a paucity of fluorescent signal, mainly from autofluorescence that is similar to what is seen in the Au50PEG nanoparticle perfused mouse cochlea (**D**).

To assess for the location of the gold nanoparticles, we performed two-photon imaging after nanoparticle infusion. A total of 2 µL of the Au50PEG nanoparticle formulation was infused into the cochlea via the PSCC, and the mouse was kept alive under anesthesia for 7 h after PSCC infusion. Then the mouse was euthanized, and the otic capsule was excised. A portion of the cochlear bone was removed at the base to allow visualization of the organ of Corti, and the cochlea was adhered to a chamber with the apical portion mounted inferiorly for simultaneous recording of optical coherence microscopy and two-photon image stacks. Optical coherence microscopy has a higher resolution than traditional OCT imaging, which allowed us to visualize structures within the organ of Corti (Figure 7A), including the outer hair cell region (rectangle), nerve fibers crossing through the tunnel of Corti (bracket), and the inner hair cell region (oval). In the two-photon fluorescent images in Au50PEG perfused cochlea, areas of fluorescence were present (arrows), especially in the red channel from the gold nanoparticles (Figure 7B). The green channel showed autofluorescence signal and bleed-through from the red channel (Figure 7C), and thus it was less reliable in localizing the nanoparticles despite the AlexaFluor488 fluorophore. Upon merging of the optical coherence microscopy and two-photon fluorescence images (Figure 7D), there clearly is a signal from the nanoparticles at the edge of the tunnel of Corti in the inner hair cell region (thick and thin arrows). This is further demonstrated in the XZ view (Figure 7E) at the cross-section of the thin arrow, which shows the tunnel of Corti (bracket) and signal from the nanoparticles in the inner hair cell region (thin arrow). There was no obvious signal along the osseus spiral lamina or endosteum of the perilymphatic duct.

In a control mouse, the microstructural elements of the organ of Corti are seen in the optical coherence microscopy scan (Figure 7F) including the outer hair cell region (rectangle), nerve fibers in the tunnel of Corti (bracket), and inner hair cell region. In the two-photon fluorescent images, there is minimal auto-fluorescence signal in the red channel (Figure 7G) and green channel (Figure 7H) when compared to the Au50PEG perfused cochlea (Figure 7B,C). The merged optical coherence microscopy and two-photon fluorescence images show a paucity of fluorescence signal in the control cochlea (Figure 7I). These data show that there is nanoparticle deposition into areas in the organ of Corti, in this case the basal portion of the inner hair cell region, which would enable therapeutic targets.

## 3. Discussion

Delivering therapeutics to the cochlea is already happening in clinical practice. Intratympanic injection of steroids into the middle ear with diffusion into the inner ear is routine treatment for sudden sensorineural hearing loss. Round window and lateral semicircular canal infusion of AAV is being performed for gene therapy clinical trials in otoferlin deficiency, which causes an auditory neuropathy spectrum phenotype that results in severe to profound sensorineural hearing loss [22,23]. Here we show that nanoparticles offer another approach to intracochlear drug delivery. We found that gold nanoparticles ranging from 52–102 nm at high 10^12^–10^13^ particles/mL concentrations can be infused via the PSCC and perfuse the entire cochlear perilymphatic space in mice without disrupting cochlear mechanics. This means that neither the mass of the nanoparticles nor the infusion technique adversely affects a key aspect of cochlear physiology, the non-linear vibratory characteristics in response to sound. Our cochlear mechanics results at 1 h after nanoparticle perfusion demonstrate that nanoparticles are a safe therapeutic delivery system when using direct inner ear infusion and pave the way perhaps for not just treating and rescuing severe to profound hearing loss, but also for treating milder forms of hearing loss and other types of inner ear disease that may not have severe hearing loss.

Direct inner ear infusion is an efficient method to deliver therapeutics into the cochlea as the drug directly enters the inner ear space [11]. Other techniques for inner ear drug delivery include intratympanic and systemic delivery methods. The round window membrane significantly limits the amount of drug in the inner ear to 1/1000 of the drug concentration delivered to the middle ear [58]. The blood labyrinth barrier also significantly limits drug entry from the bloodstream into the cochlear fluid compartments, with higher risks of systemic side effects [17]. A few studies have shown some efficacy with nanoparticle therapeutic delivery to the rodent cochlea. However, a number of these studies were performed ex vivo or attempted delivery that required the therapeutic to traverse the round window membrane [30,31,59].

We provide a method for direct intracochlear nanoparticle delivery. Though we do not deliver a therapeutic with our nanoparticles, we show that nanoparticles enter the cochlear perilymphatic space with PSCC infusion, and they eventually reach the inner hair cell region of the organ of Corti. This is where therapeutics need to reach to be effective in treating hearing loss. The mechanism by which this occurs requires further study but likely occurs via transcytosis through supporting cells in the basilar membrane. Transcytosis is a common mechanism by which nanoparticles can cross cell layers, such as in the blood brain barrier [39], and is a potential mechanism by which nanoparticles enter through the basilar membrane into the organ of Corti from the perilymphatic compartment.

Limitations of our study include the lack of long-term cochlear mechanics and hearing data. Though PSCC infusion can be performed as a survival surgery in mice, opening the bulla to expose the cochlea is too invasive to allow for survival surgery, thus limiting the amount of time that cochlear mechanics recordings can be made. We were more interested in whether any changes in cochlear mechanics would occur after gold nanoparticle infusion due to the mass of the gold. Thus, auditory brainstem responses (ABRs) as a surrogate for hearing status were not measured. However, it is reasonable to infer based on previous work that unchanged cochlear mechanics would also result in unchanged ABRs in these wild-type CBA/CaJ mice. For example, we previously showed that artificial perilymph infusion via the PSCC does not change cochlear function or auditory brainstem responses (ABR) at 1 h post-infusion in these wild-type mice, and other studies also show that tube insertion and slow PSCC infusion of 1–2 µL do not affect ABR [28,29]. Measuring ABR would be important to investigate with future work utilizing nanoparticles delivering a therapeutic, as outcomes could vary with the hearing loss model. For example, intact cochlear mechanics could still result in hearing loss on ABRs in auditory neuropathy spectrum phenotypes resulting from impaired synaptic transmission between the inner hair cell and afferent auditory nerve fibers. Lastly, the long-term deposition of nanoparticles in the inner ear, and whether some of the nanoparticles migrate to the intracranial space through the cochlear aqueduct, is a topic of future study.

Development of therapeutic strategies to treat or reverse hearing loss, and delivery of therapeutics into the inner ear, is becoming increasingly important as our knowledge of causative mechanisms for hearing loss grows. Intracochlear therapeutic delivery with PSCC nanoparticle infusion can serve this purpose. Future work can include targeting the nanoparticles to different regions in the organ of Corti or engineering them to deliver a variety or even a combination of therapeutic classes, including small molecules, proteins, and nucleic acids, which could be required to treat the multitude of etiologies for hearing loss and inner ear disease.

## 4. Materials and Methods

*Nanoparticle Synthesis.* A fluorescent AlexaFluor488-cadaverine (AF488) (ThermoFisher Scientific, Waltham, MA, USA, used as received) was conjugated via an amide bond to a functionalized polyethylene glycol (PEG) polymer PEG5k-OPSS (Creative PEGWorks, Chapel Hill, NC, USA, used as received) (Figure 1A). Excess AF488 was separated from the PEGylated AF488 using a 3 kDa filter cutoff (Millipore Amicon Ultra, Millipore Sigma, Burlington, MA, USA) in microcentrifuge at 15,000 rpm. Spherical gold nanoparticle (NP) cores of 20 nm, 50 nm, and 80 nm sizes were purchased from nanoComposix (San Diego, CA, USA, used as received) and first stirred with AlexaFluor488-PEG5k-OPSS to create a covalent disulfide linkage on the surface of the gold NPs (Figure 1B). Then, excess methoxy-PEG5k-SH (Laysan Bio, Arab, AL, USA, used as received) was added to the reaction to form covalent disulfide bonds on any further reactive binding sites on the gold NP core (Figure 1C) to stabilize the NP and prevent aggregation. The NPs were centrifuged at 15,000 rpm to form a pellet, which was washed three times to remove excess PEG5k, and resuspended in artificial perilymph (140 mM NaCl, 2 mM KCl, 2 mM MgCl_2_, 2 mM CaCl_2_, 20 mM HEPES with pH 7.4 and 303–307 mOSm/kg) at a high concentration in preparation for PSCC infusion. This created fluorescently labeled gold NPs, which are diagramed in Figure 1. The NP was stable in physiologic salt concentrations.

*Nanoparticle Characterization.* The NP size and charge of the 20 nm, 50 nm, and 80 nm gold cores containing fluorophore and mPEG, named Au20PEG, Au50PEG, and Au80PEG, were measured using a Brookhaven Instruments ZetaPALS with dynamic light scattering and zeta potential to determine hydrodynamic diameter and surface charge, respectively. Values are shown in Table 1 and are consistent with similar types of gold NPs previously reported [39,49]. We confirmed these NPs were fluorescent using a fluorimeter, epifluorescent microscope, and 2-photon fluorescent microscopy. Though NP concentration can be determined using an UV-visible absorbance technique with an Eppendorf BioSpectrometer based on the gold surface plasmon resonance using absorption and Beer’s law at the maximal absorption wavelength (size dependent, 520–550 nm), the high concentration of our nanoparticle solution in artificial perilymph is out of range of the standard curve and prevents the use of this method. Thus, we calculated the estimated concentration shown in Table 1 using the volume and concentration of gold NPs from the stock solution used in the synthesis, and the final volume of the concentrated functionalized nanoparticle solution.

*Mouse Surgery.* All experiments were performed according to protocols approved by the Institutional Animal Care and Use Committee at the University of Southern California, and all methods were performed in accordance with the relevant guidelines and regulations between 2021 and 2024. We used CBA/CaJ (JAX#: 000654, The Jackson Laboratory, Bar Harbor, ME, USA) wild-type male and female mice with normal hearing age 7–10 weeks [60,61]. Mice were anesthetized using intraperitoneal 80–100 mg/kg ketamine and 5–10 mg/kg xylazine. No invasive procedures were performed until surgical anesthesia was reached. Anesthesia depth was assessed at 15-min intervals with supplemental anesthetic doses administered to maintain anesthesia. An electric heating pad maintained the animal’s body temperature at 38–39 °C. We then proceeded with a post-auricular approach to the middle ear bulla, which was opened to reveal the otic capsule bone and round window membrane [62]. Once the experiment was completed, the mouse was euthanized while under anesthesia.

*PSCC Infusion Technique.* After opening the middle ear bulla to expose the mouse cochlea, the PSCC was exposed and the PSCC infusion procedure was completed as previously described [25,26,28]. Briefly, a canalostomy was made in the PSCC with the tip of a 28-gauge needle. The infusion tubing is a 110 µm polyimide tubing (MicroLumen, Oldsmar, FL, USA) attached inside a 280 µm polyethylene tubing (SIMS Portex Ltd., Hythe, Kent, UK) with Krazy Glue, which was placed over the needle of a 10 µL gastight Hamilton syringe (Hamilton, Reno, NV, USA). The infusion tubing was filled with gold nanoparticle solution at the concentration listed in Table 1 suspending in artificial perilymph (140 mM NaCl, 2 mM KCl, 2 mM MgCl_2_, 2 mM CaCl_2_, 20 mM HEPES with pH 7.4 and 303–307 mOSm/kg). This was a high concentration of gold nanoparticles based on what has previously been used to visualize nanoparticles within the cochlea using OCT [28,63]. The infusion tubing containing the gold nanoparticle solution was placed into the PSCC canalostomy and was sealed with histoacryl tissue glue (Tissue Seal, LLC, Ann Arbor, MI, USA). 1 µL of nanoparticle solution in artificial perilymph was infused into the PSCC at 0.5 µL/min over 2 min using a one-channel programmable syringe pump (New Era Pump Systems, Inc., Farmingdale, NY, USA). For two-photon imaging experiments, another 1 µL was delivered 10 min after the initial infusion to increase the concentration of nanoparticles delivered for fluorescence visualization and minimize infusion volume and pressure at a single time point.

*Optical Coherence Tomography and Vibrometry Measurements.* Our custom-built OCT system has been previously described [60,63,64]. We have built multiple versions of this system. The one used for this work had a swept-laser source with a center wavelength of 1306 nm, 90.4 nm bandwidth, and sweeping at 100 kHz. The resulting axial resolution, assuming a refractive index of 1.4 and a Hann window for spectral shaping, was 13.5 µm. The sample arm of the OCT interferometer is integrated into a stereo microscope, Zeiss Stemi-2000. A 2D voice-coil mirror setup for telecentric scanning through an objective lens provided a lateral resolution of 9.8 µm.

OCT images were obtained at baseline after exposing the cochlea, after infusion tubing insertion into the PSCC, during and at time intervals immediately after PSCC infusion up to approximately 60–75 min after nanoparticle infusion. Sound stimuli for vibrometry measurements were delivered in a similar manner as previously described in an open field configuration using an Etymotic ER2SE earbud and consisted of pure tones with frequencies ranging from 0.5–15 kHz in 0.5 kHz steps and levels ranging from 10–80 dB SPL in 10 dB steps. Each stimulus duration was 100 ms. Basilar membrane vibrometry measurements were taken at baseline following surgical opening of the bulla, after performing a PSCC canalostomy and inserting tubing containing nanoparticle solution, immediately following infusion of the nanoparticle solution, up to approximately 60–75 min after NP infusion, and immediately following euthanasia of the mouse at the conclusion of the experiment. The characteristic frequency (CF) was identified as the frequency with the largest vibratory response to the lowest stimulus intensity that produced a vibratory response above the noise floor. Cochlear gain was calculated as the difference in vibratory sensitivity (displacement normalized to the sound intensity) to 80- and 20-dB SPL stimuli at the CF. Q_10dB_ was calculated at 40 dB SPL. The gain, CF, and Q_10dB_ were calculated from basilar membrane vibrometry recordings prior to (0 min), after PSCC infusion of the solution (at approximately 5–15 min referred to as immediately after infusion and 60–75 min referred to as 1 h after infusion), and after euthanasia of the mouse. These data were analyzed in MATLAB. All vibrometry experiments were carried out on the left ear.

*Two-Photon Optical Coherence Microscopy (2P OCM).* After PSCC infusion of Au50PEG nanoparticles, the mouse was kept under anesthesia for 7 h and then euthanized. The cochlea was excised, and the otic capsule bone was scraped away to be able to visualize the organ of Corti directly. The freshly excised cochlea was held in place on a homemade glass-bottom chamber with Histoacryl glue (Tissue Seal, LLC, Ann Arbor, MI, USA) and imaged using our two-photon optical coherence microscopy system detailed below. These were compared with cochlea from control mice.

Our two-photon and optical coherence microscopy system has previously been described [65,66]. Briefly, two-photon excitation was accomplished by scanning a 150-mW femtosecond pulsed laser at 930 nm (Chameleon Ultra II, Coherent, Saxonburg, PA, USA) across the sample through the trinocular port on a Nikon upright microscope (Nikon Instruments Inc., Melville, NY, USA) with a Nikon 16× (0.8 NA) water-immersion objective. Fluorescence from the sample was collected through the objective and separated from the excitation beam path with a long-pass dichroic mirror with a cutoff wavelength of 735 nm. The red and green fluorescence channels were separated using another long-pass dichroic mirror with a cutoff wavelength of 585 nm. A final filter was added to the green channel that further reduced the spectrum of green fluorescence to a bandwidth of 40 nm centered at 500 nm. The detectors used in both channels were hybrid photodetectors (R11322U-40, Hamamatsu, Japan) with variable gain settings that allowed control of relative brightness between the fluorescence detected from the two channels.

Two-photon and optical microscopy z-stacks were acquired simultaneously with customized software. The field of view was 134 μm in diameter. Volumes were acquired using 1 µm steps in the z direction for a total of 50 steps. Images were imported into ImageJ for analysis.

*Two-Photon Microscopy*. Gold nanoparticle solution on a slide was imaged using an inverted microscope (Leica SP-8X MP, Leica Microsystems, Deerfield, IL, USA) with an excitation wavelength of 930 nm and emission wavelengths of 494–529 nm and 588–642 nm for the green and red channels, respectively. A 20× (0.75 N.A.) objective was used for imaging. The images were exported into ImageJ for analysis.

## 5. Conclusions

PSCC infusion is an efficient method for direct inner ear delivery of nanoparticles. The nanoparticles initially enter the perilymphatic space and eventually deposit within the cochlea in the inner hair cell region. Despite the mass of gold nanoparticles, infusion and delivery of these nanoparticles do not cause any changes in cochlear mechanics at the basilar membrane.

## Figures and Tables

**Table 1:** Functionalized gold nanoparticle characteristics. Size, polydispersity index, zeta potential, calculated concentration, and gold mass concentration of the formulated nanoparticles Au20PEG, Au50PEG, and Au80PEG, with 20 nm, 50 nm, and 80 nm spherical gold cores, respectively, containing AlexaFluor488 fluorophore and polyethylene glycol (PEG) for stabilization in physiologic salt solutions.

Nanoparticle Formulation: Core Size and Functionalization (Abbreviation)	Size (nm) ± Std Error	Polydispersity Index	Zeta Potential (mV)	Concentration (Particles/mL)	Gold Mass Concentration (mg/mL)
20nm AF488-AuNP mPEG (Au20PEG)	52.3 ± 0.7	0.268	−10.53 ± 2.06	7.8 × 10^13^	5.3
50nm AF488-AuNP mPEG (Au50PEG)	83.1 ± 2.0	0.164	−12.46 ± 1.27	3.7 × 10^12^	5.3
80nm AF488-AuNP mPEG (Au80PEG)	101.8 ± 1.0	0.158	−1.71 ± 1.40	1.1 × 10^12^	5.3

**Table 2:** Cochlear mechanics are unchanged after gold nanoparticle infusion via PSCC. Best frequency, gain from 20 dB to 80 dB SPL, and Q_10dB_ at 40 dB mean values for *n =* 5 mice, calculated for each nanoparticle formulation Au20PEG, Au50PEG, and Au80PEG at baseline, after canalostomy, right after, and 1h after PSCC nanoparticle infusion. There are no statistically significant differences in these cochlear mechanics parameters at any condition compared to baseline on paired *t*-test.

Nanoparticle Formulation, Time Point	Best Frequency (Mean ± SD) (kHz)	*p*-Value (re Baseline)	Gain (20 to 80 dB SPL) (Mean ± SD)	*p*-Value (re Baseline)	Q_10dB_ (at 40 dB SPL) (Mean ± SD)	*p*-Value (re Baseline)
Au20PEG (*n =* 5)						
Baseline	8.5 ± 1.0		37.8 ± 6.4		2.35 ± 0.27	
Canalostomy	9.0 ± 0.5	*p* = 0.19	36.3 ± 4.8	*p* = 0.6	2.27 ± 0.36	*p* = 0.66
Right After	9.0 ± 0.8	*p* = 0.14	36.1 ± 5.2	*p* = 0.4	2.31 ± 0.50	*p* = 0.77
1 h After	8.5 ± 1.0	*p* = 1.0	32.3 ± 7.3	*p* = 0.10	2.20 ± 0.51	*p* = 0.58
Au50PEG (*n =* 5)						
Baseline	8.3 ± 0.6		43.8 ± 4.2		1.98 ± 0.34	
Canalostomy	8.3 ± 0.6	*p* = 1.0	41.2 ± 8.0	*p* = 0.32	1.90 ± 0.37	*p* = 0.69
Right After	8.2 ± 0.4	*p* = 0.62	37.7 ± 9.0	*p* = 0.13	2.05 ± 0.51	*p* = 0.75
1 h After	8.4 ± 0.9	*p* = 0.86	36.9 ± 10.1	*p* = 0.27	2.17 ± 0.48	*p* = 0.41
Au80PEG (*n =* 5)						
Baseline	8.0 ± 0.5		41.4 ± 6.7		2.38 ± 0.84	
Canalostomy	8.2 ± 1.0	*p* = 0.76	39.7 ± 9.3	*p* = 0.80	1.99 ± 0.45	*p* = 0.22
Right After	8.6 ± 0.7	*p* = 0.18	41.6 ± 6.4	*p* = 0.96	2.36 ± 0.40	*p* = 0.97
1 h After	8.7 ± 1.0	*p* = 0.23	43.1 ± 4.8	*p* = 0.65	2.04 ± 0.64	*p* = 0.32

## Data Availability

Data supporting the findings of this study will be shared upon request by emailing the first author at dorothy.pan@med.usc.edu. We also have uploaded key data sets to our GitHub site https://github.com/jso111/ (accessed on 15 November 2024).

## Supplementary Materials

The following supporting information (Supplementary Video S1) can be downloaded at: https://www.mdpi.com/article/10.3390/ijms26010126/s1.
